# *Kjellmaniella crassifolia* for the Prevention of Ultraviolet B-Induced Oxidative Stress in HaCaT Keratinocytes

**DOI:** 10.4014/jmb.2508.08041

**Published:** 2025-11-27

**Authors:** Kirinde Gedara Isuru Sandanuwan Kirindage, Arachchige Maheshika Kumari Jayasinghe, Hyun-Jung Yun, Mi-Soon Jang, Kunbawui Park, Bomi Ryu, Jimin Hyun, Ginnae Ahn, Jae-Young Oh

**Affiliations:** 1 Department of Food Technology and Nutrition, Chonnam National University, Yeosu 59626, Republic of Korea; 2 Food Safety and Processing Research Division, National Institute of Fisheries Science, Busan 46083, Republic of Korea; 3 Research Cooperation Division, National Institute of Fisheries Science, Busan 46083, Republic of Korea; 4 Major of Food Science and Nutrition, Pukyong National University, Busan 48513, Republic of Korea

**Keywords:** *Kjellmaniella crassifolia*, photoprotection reactive oxygen species, apoptosis, MAPK signaling, Nrf2/HO-1 activation

## Abstract

Ultraviolet B (UVB) radiation induces oxidative stress, contributing to skin damage and elevating the risk of chronic skin disorders. In response to growing interest in natural alternatives to synthetic compounds, this study evaluated the photoprotective effects of *Kjellmaniella crassifolia* ethanol extract (KCE) in UVB-exposed HaCaT keratinocytes. The cytotoxicity/cytoprotective activity of KCE was assessed using MTT, and DCF-DA assays to measure cell viability and intracellular reactive oxygen species (ROS), respectively. Apoptotic cell death was examined through nuclear staining, Annexin V/PI assays, and mitochondrial membrane potential analysis via JC-1 staining. Western blot results indicated that KCE suppressed the mitochondrial-mediated apoptotic pathway, reduced phosphorylation of MAPK and NF-κB, and activated the Nrf2/HO-1 antioxidant signaling pathway. Overall, these findings demonstrate that KCE effectively attenuates UVB-induced oxidative stress and apoptosis, highlighting its potential as a natural bioactive ingredient for skin protection in cosmeceutical applications.

## Introduction

With industrialization, ozone layer depletion allows more ultraviolet radiation (UVR) to penetrate the surface of the Earth, which has been confirmed by comparing UVR measurements at many clean-air sites [1]. Ultraviolet B (UVB) is a type of ultraviolet light (280–315 nm) that leads to more significant damage to the skin rather than providing additional benefits to living organisms [2]. This damage includes sunburn, premature aging, and increased risk of skin cancer. UVB radiation can alter DNA in skin cells, leading to mutations such as DNA lesions. Even though these alterations are removed by the nucleotide excision repair pathway, some mutations can disrupt normal cell function and potentially result in cancerous growth [3, 4]. The World Health Orgasnization announced in 2022 that one in every three cancers is diagnosed as some form of skin cancer [5]. As reported, over 120,000 skin cancer-associated deaths were confirmed from over 1.5 million cases of skin cancer globally [5].

Moreover, the direct absorption of UVB photons by endogenous photosensitizer molecules reacts with oxygen and generates free radicals in the skin [6]. It increases oxidative stress in skin cells, resulting from an imbalance between reactive oxygen species (ROS) and antioxidant mechanisms in cells. This imbalance leads to the accumulation of ROS, which can damage cellular components like DNA, proteins, and lipids [7]. Oxidative stress contributes to skin aging, characterized by wrinkles, loss of elasticity, and uneven pigmentation. It also plays a role in the development of skin diseases, including cancer [8]. Moreover, ROS-induced damage can trigger inflammatory responses and disrupt normal cell signaling pathways [9].

Oxidative stress in epidermal cells triggers key signaling pathways that regulate apoptosis and cell cycle arrest. Mitochondrial dysfunction releases cytochrome c, activating caspases and programmed cell death [10]. Concurrently, ROS activate NF-κB, promoting pro-inflammatory cytokine expression, and MAPKs, altering gene expression to affect growth, differentiation, and survival [9, 11]. Together, these pathways contribute to skin damage, inflammation, and disease progression.

Naturally available bioresources have gained attention due to sustainability and safety in use as cosmeceuticals to prevent/recover skin damage. Among them, seaweeds are spotlighted since rich in bioactive compounds, including antioxidants, polyphenols, and vitamins [12]. These compounds help prevent oxidative stress, a key factor in skin aging and damage. Reportedly, seaweed extracts can enhance the natural defense mechanisms of the skin by boosting cellular repair processes and reducing inflammation [13]. Previous researchers concluded that incorporating seaweed extracts into cosmeceuticals can develop products with protective and reparative benefits, promoting healthier and more resilient skin [9, 12].

Crude extracts offer advantages over single compounds in skin treatments due to their complex mixture of bioactive molecules, which can act synergistically to provide antioxidant, anti-inflammatory, and antimicrobial effects. Pharmacologically, this multi-target approach addresses several pathways simultaneously, enhancing prevention and repair of UV-induced skin damage. Crude extracts are often safer, with fewer side effects, and their cost-effective production increases accessibility. They also support sustainable, eco-friendly practices. Previous reports have described antioxidants or anti-inflammatory effects of *K. crassifolia*-derived polysaccharides and other organic solvent extracts [14, 15]; however, the specific contribution of ethanol-soluble components and their mechanistic involvement in UVB-induced skin cell protection have not been characterized. To address this gap, the present study elucidates the photoprotective mechanism of 70% ethanol extract of *K. crassifolia* (KCE), emphasizing its modulation of the Nrf2/HO-1 and MAPK/NF-κB signaling pathways in UVB-exposed HaCaT keratinocytes.

## Materials and Methods

### Materials

Dulbecco’s modified eagle medium (DMEM), and penicillin/streptomycin mixture were purchased from GibcoBRL (USA). Fetal bovine serum (FBS) was purchased from Welgene (Republic of Korea). Folin and Ciocalteu’s phenol reagent, bovine serum albumin (BSA), gallic acid, 2,2- diphenyl-1-picrylhydrazyl (DPPH), 2,2-azino-bis (3-ethylbenzothiazoline-6-sulfonic acid) diammonium salt (ABTS), 3-(4,5- dimethylethylthiazol-2-yl)-2,5-diphenyltetrazolium bromide (MTT), Dimethyl sulfoxide (DMSO), 2’7’-dichlorodihydrofluorescein diacetate (DCF-DA), ethidium bromide (EB), acridine orange (AO), Hoechst 33342, propidium iodide (PI), and ascorbic acid (AA) were purchased from Sigma-Aldrich (USA). D-glucose was purchased from Junsei Chemical Co., Ltd.(Japan). JC-1 assay kit was acquired from Thermo Fisher Scientific (USA), and the Annexin V assay kit was from Invitrogen (USA). NE-PER nuclear and cytoplasmic extraction kit, Pierce RIPA buffer, BCA protein assay kit, polyvinylidene fluoride (PVDF), 1-step transfer buffer, SuperSignal West Femto Maximum Sensitivity Substrate and PageRuler Plus pre-stained protein ladder was obtained from Thermo Fisher Scientific. Required antibodies for western blot analysis were purchased from Cell Signaling Technology Inc. (USA) and Santa Cruz Biotechnology Inc. (USA). Skim milk powder was obtained from BD Difco (USA). The remaining solvents and reagents were of analytical grade.

### Sample Collection and Extraction

*K. crassifolia* was collected from the southern coast of the Korean peninsula. Extraction was performed following a previously reported method with slight modifications [16]. Briefly, the seaweed was thoroughly rinsed with running water to remove sand and surface debris. Following that, the samples were air-dried at room temperature and ground into a fine powder using an IKA MF10 laboratory pulverizer (Germany). The powdered material was then extracted with 70% ethanol at a 1:50 (w/v) ratio by incubating it overnight in a hot water shaker at 40°C. After 24 h, the mixture was filtered using a vacuum filtration system, and the filtrate was concentrated by rotary evaporation to yield the crude ethanol extract (KCE). The dried extract was stored at −20°C for use in subsequent experiments.

### Compositional Analysis of the Crude Extract of KCE

The total phenolic content, total protein content, and total carbohydrate content were evaluated as described in a previous publication [16]. In brief, total phenolic content was determined using the Folin–Ciocalteu method, with gallic acid as the standard, following incubation of the crude extract with the reagent in the dark. Total protein content was quantified using the Lowry method, employing bovine serum albumin (BSA) as the standard. Total carbohydrate content was measured using the phenol–sulfuric acid method, with a standard curve generated from a glucose series. The UPLC–QTOF–MS/MS analysis of the KEC is listed in Supplementary File S1.

### Radical Absorbance Capacity of KCE

DPPH and ABTS^+^ radical scavenging activities were evaluated according to the method described in a previous publication [17]. For the DPPH radical scavenging assay, 100 μl of 150 μM DPPH solution was mixed with 100 μl of KCE (250 μg/ml) and incubated in the dark at room temperature for 30 min. The absorbance was then measured at 517 nm using a SpectraMax M2 microplate reader (Molecular Devices, USA). For the ABTS^+^ assay, 50 μl of the ABTS_•_^+^ working solution—prepared by reacting ABTS with potassium persulfate—was added to KCE (250 μg/ml) and incubated in the dark for 10 min. Absorbance was measured at 414 nm.

### Cell Culture

HaCaT keratinocytes were cultured in DMEM media supplemented with 10% heat-inactivated FBS and 1%penicillin/streptomycin mixture. Cells were maintained under a controlled humidified environment at 37°C with 5% CO_2_ and sub-cultured once in 2 days until exponential growth was preferable for cell seeding. Cells in their exponential growth were used for further experiments.

### Cell Viability and Intracellular ROS Production Analysis

The following experiment was done using a method in a previous study with slight modifications [9]. Briefly, HaCaT keratinocytes were seeded at a density of 1 × 10^4^ cells/well in a 96-well plate and pretreated with varying concentrations of KCE (31.3, 62.5, 125, and 250 μg/ml) for 2 h. Following pretreatment, the cell culture medium in all wells was transferred into a labeled 96-well plate and replaced with 200 μl phosphate-buffered saline (PBS) in each well. Cells were then exposed to UVB irradiation (40 mJ/cm^2^) using an ultraviolet cross-linker (Vilber BLX-254, France). The UVB intensity was calibrated prior to experiments to ensure accurate energy delivery, and the cross-linker was positioned to provide uniform irradiation across the plate surface. Immediately after UVB exposure, PBS was gently removed and replaced with the previous culture medium kept in the labeled 96-well plate. After a 1-hour incubation, intracellular reactive oxygen species (ROS) levels were assessed using the DCF-DA assay. Fluorescence intensity was measured with a SpectraMax M2 microplate reader at an excitation wavelength of 485 nm and emission at 528 nm. UVB-exposed cells were analyzed using the MTT assay after 24 h of incubation to evaluate the cytoprotective effect of the KCE samples following a pre-described method in a previous research paper [17]. DCF-DA-stained HaCaT keratinocytes were further analyzed by CytoFLEX flow cytometer (Beckman Coulter, USA). Selected KCE concentrations were used for further experiments.

### Evaluation of Early and Late Apoptosis

Morphological changes of early and late apoptosis were examined by Acridine orange (AO) and ethidium bromide (EB) staining. Cells were seeded in 24-well plates with 1×10^5^ cells per well. After 24 h incubation, cells were treated with different concentrations of KCE and exposed to UVB radiation following the method described in the ‘Cell Viability and Intracellular ROS Production Analysis’ section. Afterward, cells were treated with the mixture of AO/EB (10 μl) and incubated for another 10 min. Cell morphological changes were examined by using the EVOS M5000 imaging fluorescence microscope (Thermo Fisher Scientific).

### Evaluation of Apoptotic Body Formation

Hoechst 33342 (0.5 mg/ml) and PI (2.5 μM) staining assay was conducted to evaluate the apoptotic body formation outlined in the previous study [18]. In brief, HaCaT cells were seeded at a density of 1 × 10^5^ cells/well in 24-well plates and incubated for 24 h. Cells were then treated with KCE for 1 h before UVB exposure. Following UVB irradiation, cells were incubated for additional 24 h as described in the ‘Cell Viability and Intracellular ROS Production Analysis’ section. For nuclear staining, 10 μl of Hoechst dye was added to each well, and cells were incubated for 10 min. Nuclear morphology was observed and imaged using an EVOS M5000 fluorescence microscope.

### Flow Cytometric Analysis of Annexin V and JC-1 Assay

Cell culture, sample treatment, and stimulation were same as the methos described in the ‘Cell Viability and Intracellular ROS Production Analysis’ section. The Annexin V assay was performed using the Annexin V-FITC/PI Apoptosis Detection Kit (Invitrogen, Cat. #V13242). Briefly, cells were harvested 6 h after UVB exposure and transferred to light-protected microcentrifuge tubes. Staining was carried out according to the manufacturer's instructions, followed by analysis using a CytoFLEX flow cytometer. Mitochondrial membrane depolarization was assessed using the MitoProbe JC-1 Assay Kit (Invitrogen), following 4 h of incubation after UVB exposure. The assay was conducted following the manufacturer’s protocol, and fluorescence was analyzed using the CytoFLEX flow cytometer.

### Cell Cycle Analysis

Cell cycle analysis was conducted according to the method described in one of our previous studies [18]. In brief, after being incubated for 24 h following UVB irradiation, the cells were harvested, washed with PBS, and allowed to permeabilize for 30 min in 70% ethanol. The cells were then resuspended with PBS containing EDTA, PI, and RNase. The cell cycle was analyzed by using the CytoFLEX flow cytometer.

### Western Blot Analysis

Following UVB exposure, cells were incubated for the designated times specific to each signaling pathway, then harvested and lysed with the NE-PER nuclear and cytoplasmic extraction kit to separate nuclear and cytoplasmic proteins. Protein concentrations in the lysates were measured by using a BCA protein assay kit. Further steps of the experiment were carried out following the method mentioned in the previous publication [11].

### Statistical Analysis

Statistical analyses were performed using GraphPad prism version 10. All experiments were conducted in triplicate (*n* = 3), and data are presented as mean ± standard deviation (SD). One-way analysis of variance (ANOVA) was applied to determine overall differences between groups. When ANOVA indicated significance, Tukey’s honestly significant difference (HSD) post hoc test was used for multiple pairwise comparisons. A significance threshold of *p* < 0.05 was applied for all analyses. Prior to ANOVA, data were checked for normality and homogeneity of variances by using MiniTab statistical package 17 to ensure the validity of parametric testing.

## Results

### Extraction Yield and Proximate Composition of KCE

The extraction yield of KCE was 25.00 ± 0.13% relative to the initial dry weight of the sample. Among the analyzed components, carbohydrates were the most abundant, constituting 5.48 ± 0.21% of the extract. This was followed by crude protein at 4.77 ± 0.16% and total phenolic content at 2.13 ± 0.01% (Table 1).

### Antioxidant Activities of KCE

**Fig. 1:**
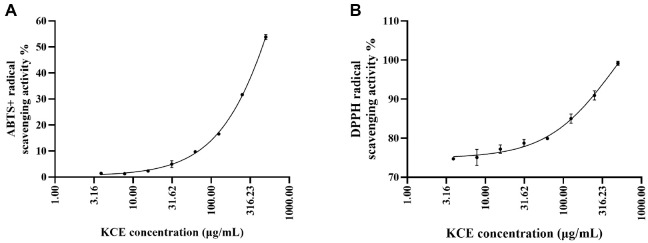
Dose-dependent ABTS^+^ (A) and DPPH (B) radical scavenging activities of *Kjellmaniella crassifolia* ethanol extract (KCE). Activities were analyzed using a three-parameter inhibitor vs. response model. Data points represent mean responses, and fitted curves show excellent agreement with experimental results. Error bars indicate standard deviation.

The antioxidant potential of the KCE was evaluated using both DPPH and ABTS radical scavenging assays across concentration ranges of 3.9-500 μg/ml. For DPPH indicated in Fig. 1B, nonlinear regression analysis with a three-parameter dose–response model revealed an IC_50_ value of 456.8 μg/ml (95% CI: 305.5–745.8 μg/ml), with a strong goodness of fit (R^2^ = 0.9834). In contrast, the ABTS assay yielded a higher IC_50_ of 1342 μg/ml (95% CI: 1090–1714 μg/ml) (Fig. 1A), with an excellent model fit (R^2^ = 0.9987). Collectively, these results demonstrate that the extract possesses dose-dependent radical scavenging activity, with greater efficacy observed against DPPH compared to ABTS radicals.

### KCE Suppressed the Oxidative Stress and Increased the Cell Viability in UVB-Exposed HaCaT Keratinocytes

**Fig. 2:**
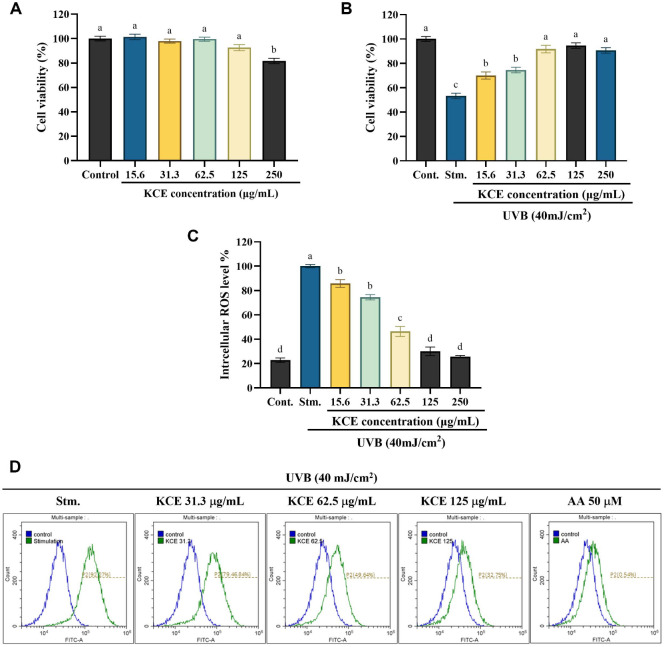
Effects of *Kjellmaniella crassifolia* ethanol extract (KCE) on HaCaT keratinocytes. (**A**) Cytotoxicity of KCE and (**B**) its impact on cell viability following UVB exposure. Intracellular ROS production was assessed using (**C**) fluorometric and (**D**) flow cytometric analyses. All experiments were performed in triplicate (*n* = 3) and data are presented as mean ± SD. Columns labeled with different letters indicate statistically significant differences (*p* < 0.05). Stm. refers to the nontreated/ negative control group.

The MTT assay results (Fig. 2A) demonstrated that KCE exhibited no significant cytotoxicity in HaCaT keratinocytes at concentrations up to 125 μg/ml. Furthermore, KCE treatment significantly enhanced cell viability in a dose-dependent manner within the 15.6–125 μg/ml range (Fig. 2B). In addition, intracellular reactive oxygen species (ROS) levels were markedly reduced by KCE in a concentration-dependent manner, as shown in Fig. 2C and 2D. These findings indicate that KCE promotes cell viability and effectively alleviates oxidative stress by suppressing intracellular ROS production in UVB-exposed HaCaT keratinocytes.

### KCE Reduced the Number of Apoptotic Events and Sub-G1 Cell Population in UVB-Exposed HaCaT Keratinocytes

**Fig. 3:**
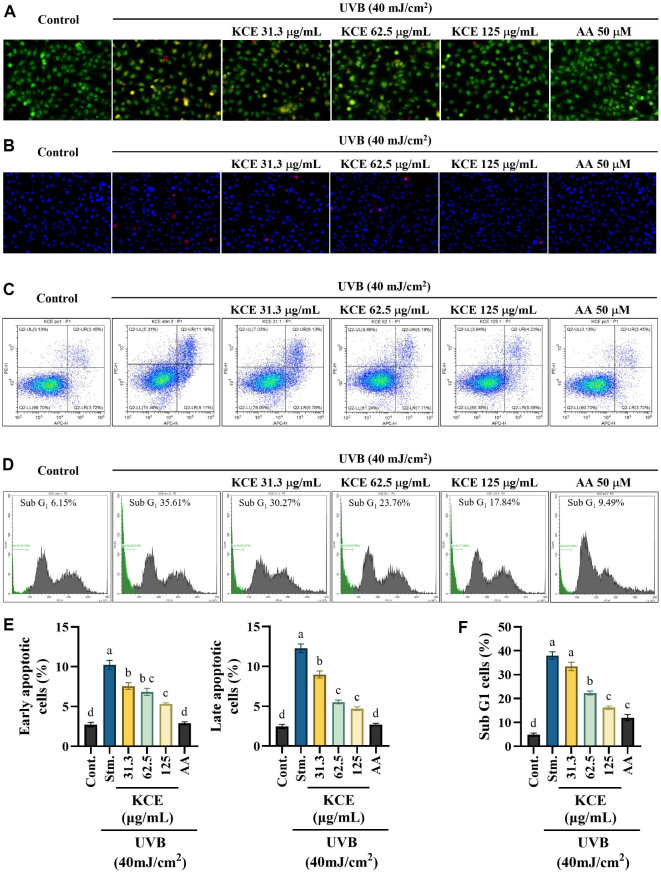
Analysis of apoptotic events in UVB-exposed HaCaT keratinocytes treated with *Kjellmaniella crassifolia* ethanol extract (KCE). Apoptosis was assessed using fluorescence microscopy with (**A**) AO/EB nuclear staining and (**B**) Hoechst/PI double staining, as well as flow cytometry with (**C**) Annexin V/PI staining and (**D**) sub-G_1_ cell population analysis. Quantitative evaluations are shown for (**E**) early and late apoptotic cell percentages and (**F**) sub-G_1_ cell accumulation. All experiments were performed in triplicate (*n* = 3) and data are presented as mean ± SD. Stm. refers to the nontreated/ negative control group.

Nuclear double staining with AO/EB indicated the early and late stages of apoptosis and necrotic cells [19]. AO/EB double staining distinguished apoptotic stages in HaCaT keratinocytes, with early apoptotic cells exhibiting yellow-green fluorescence and late apoptotic cells showing an orange hue. As shown in Fig. 3A, UVB irradiation significantly increased both early and late apoptotic cell populations. However, treatment with KCE reduced these populations in a dose-dependent manner. Similarly, nuclear staining with Hoechst 33342 and propidium iodide (PI) revealed classical apoptotic features, including chromatin condensation, nuclear fragmentation, and necrotic cells (red fluorescence) in the UVB-treated group (Fig. 3B). These features were progressively diminished with increasing concentrations of KCE, with 125 μg/ml producing effects comparable to those of the ascorbic acid (AA) positive control. The Annexin V/PI assay further confirmed the anti-apoptotic activity of KCE. UVB exposure significantly increased early and late apoptotic populations and decreased the number of viable cells (Fig. 3C). Treatment with 125 μg/ml of KCE reduced early and late apoptotic cells to 5.58% and 4.23%, respectively. In parallel, cell cycle analysis revealed a substantial increase in the Sub-G_1_ population in the UVB-exposed group (35.61%) compared to the control (6.15%) (Fig. 3D). KCE treatment significantly decreased Sub-G_1_ accumulation in a dose-dependent manner, with the highest concentration (125 μg/ml) reducing it to 17.84%. Quantitative evaluations of early and late apoptotic cell percentages (Fig. 3E) and sub-G_1_ cell accumulation (Fig. 3F) indicate the dose-dependent effect of KCE. Collectively, these findings demonstrate that KCE effectively protects HaCaT keratinocytes against UVB-induced apoptosis by reducing apoptotic and necrotic cell populations and preserving overall cell viability.

### KCE Suppressed the Mitochondria-Mediated Apoptosis in UVB-Exposed HaCaT Keratinocytes

**Fig. 4:**
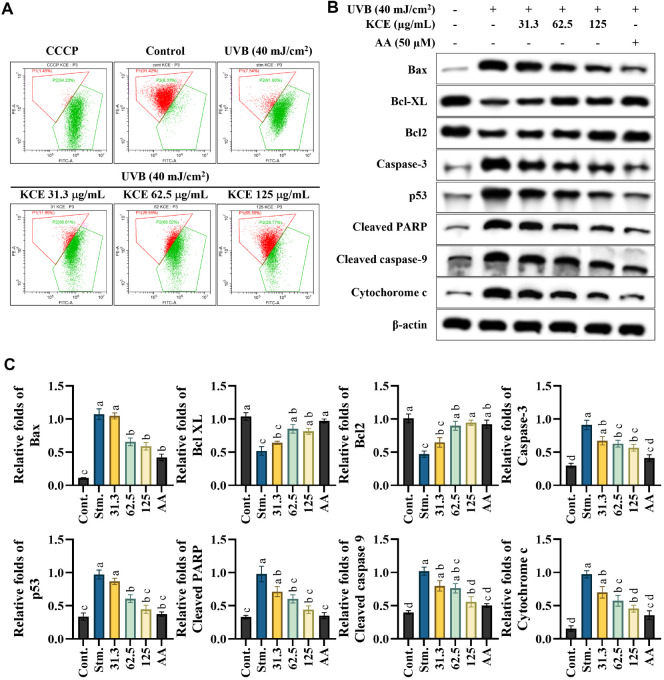
Evaluation of mitochondrial-mediated apoptosis in UVB-exposed HaCaT keratinocytes treated with *Kjellmaniella crassifolia* ethanol extract (KCE). (**A**) Mitochondrial membrane potential was assessed using the JC-1 assay. Depolarized mitochondrial membranes are shown in green, and polarized membranes are shown in red. (**B**) Western blot analysis of proteins involved in the mitochondria-mediated apoptotic pathway, with (**C**) quantification of relative protein expression normalized to β-actin. All experiments were performed in triplicate (*n* = 3) and data are presented as mean ± SD. Stm. refers to the non-treated/negative control group.

Mitochondrial health was evaluated using the JC-1 assay, with carbonyl cyanide m-chlorophenyl hydrazone (CCCP) employed as a positive control for mitochondrial depolarization. The JC-1 dye accumulates in healthy mitochondria with intact membrane potential (ΔΨm), forming aggregates that emit red fluorescence. In contrast, mitochondrial dysfunction or early apoptosis leads to depolarization of the inner mitochondrial membrane, causing JC-1 to remain in its monomeric form, which emits green fluorescence. As shown in Fig. 4A, UVB-exposed HaCaT keratinocytes displayed a fluorescence pattern indicative of mitochondrial depolarization-characterized by increased green and decreased red fluorescence-similar to the CCCP-treated group. However, treatment with KCE restored red fluorescence in a dose-dependent manner, with the 125 μg/ml concentration showing a pattern comparable to the untreated control group. This suggests that KCE effectively restores mitochondrial membrane potential in UVB-damaged cells. Mitochondrial depolarization is a key trigger of the intrinsic apoptotic pathway, initiating the release of cytochrome c and the activation of downstream apoptotic proteins. KCE treatment dose-dependently inhibited cytochrome c release and modulated apoptosis-related protein expression. Specifically, KCE upregulated anti-apoptotic proteins Bcl-2 and Bcl-xL, while downregulating pro-apoptotic proteins including Bax, cleaved caspase-9, cleaved caspase-3, cleaved PARP, and p53 (Fig. 4B). Statistical analysis of the effect of KCE on apoptotic signaling is presented in Fig. 4C. Collectively, these results demonstrate that KCE protects HaCaT keratinocytes from UVB-induced mitochondrial dysfunction and apoptosis by preserving mitochondrial membrane integrity and modulating key ragulators of the mitochondrial-mediated apoptotic pathway.

### KCE Downregulated the MAPK and NF-κB Signaling in UVB-Exposed HaCaT Keratinocytes

**Fig. 5:**
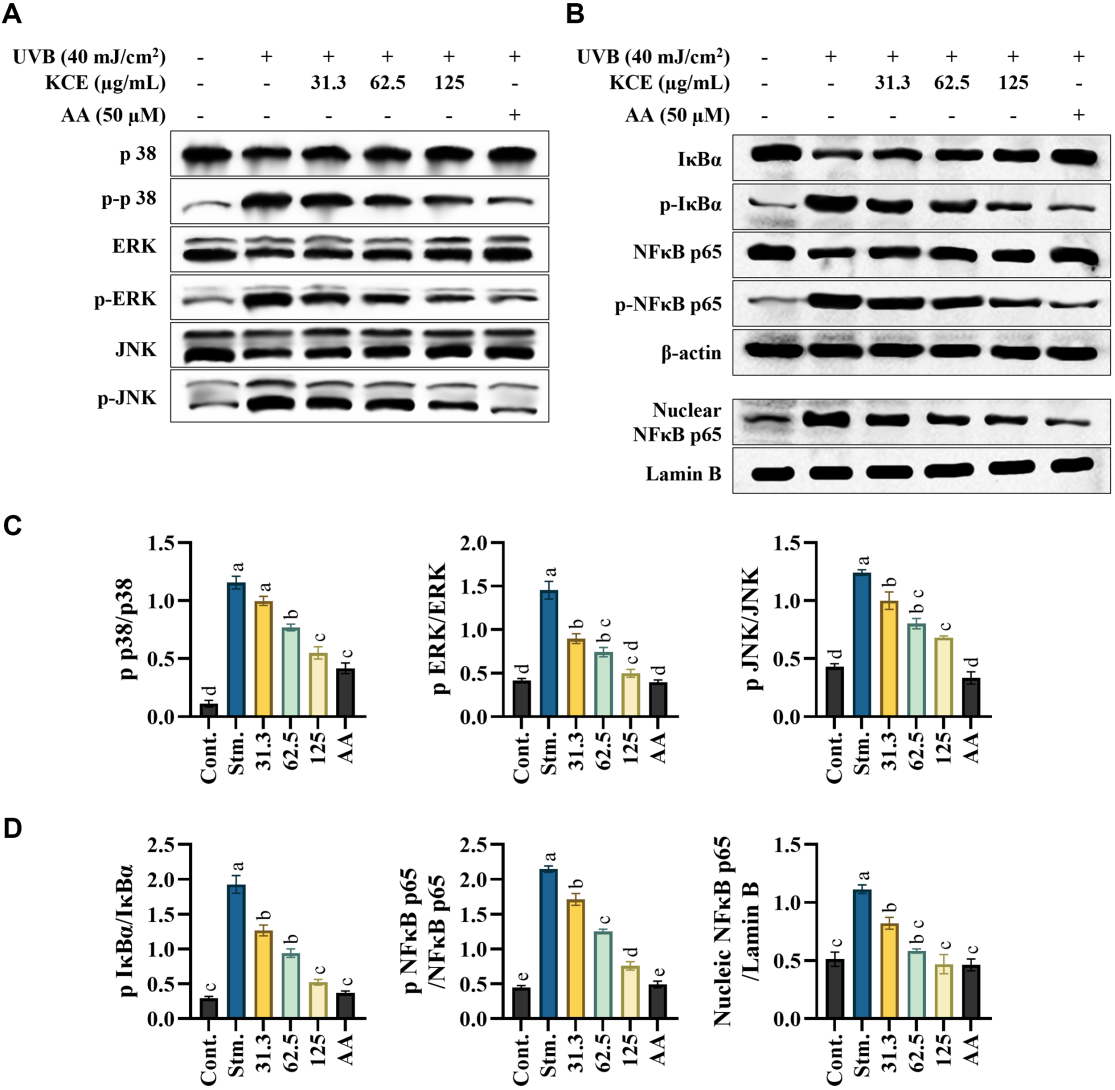
Effects of *Kjellmaniella crassifolia* ethanol extract (KCE) on MAPK and NF-κB signaling in UVBexposed HaCaT keratinocytes. (**A**) Western blot analysis of MAPK pathway proteins and (**B**) NF-κB pathway proteins. Quantitative analysis of (**C**) phosphorylated p38, ERK, and JNK relative to their total protein levels, and (**D**) phosphorylated IκBα and NF-κB p65 relative to total protein, along with nuclear NF-κB p65 normalized to lamin B. All experiments were performed in triplicate (*n* = 3) and data are presented as mean ± SD.

The MAPK and NF-κB signaling pathways are key regulators of the inflammatory response in UVB-exposed HaCaT keratinocytes. UVB irradiation significantly increased the phosphorylation of MAPK pathway components including p38, JNK, and ERK, as well as NF-κB pathway mediators such as IκBα and NF-κB p65. However, treatment with KCE effectively suppressed the UVB-induced phosphorylation of these signaling molecules in a dose-dependent manner (Fig. 5A and 5B). Relative folds of MAPK (Fig. 5C) and NF-κB signaling (Fig. 5D) indicate the quantitative changes following KCE treatment. These findings indicate that KCE mitigates UVB-triggered inflammation by inhibiting both the MAPK and NF-κB signaling path-ways.

### KCE Activated Nrf2/HO1 Signaling Pathway in UVB-Exposed HaCaT Keratinocytes

**Fig. 6:**
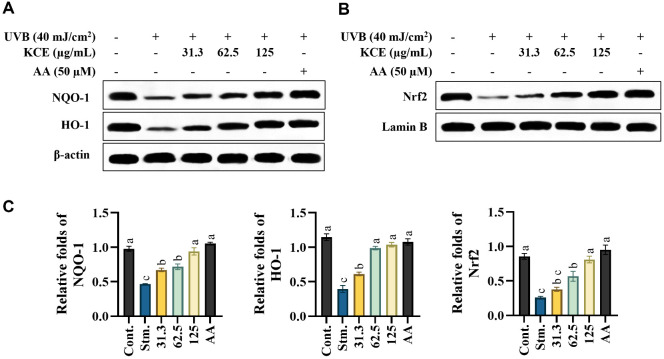
Effects of *Kjellmaniella crassifolia* ethanol extract (KCE) on Nrf2/HO-1 antioxidant signaling in UVB-exposed HaCaT keratinocytes. (**A**) Expression of HO-1 and NQO1 proteins. (**B**) Nuclear translocation of Nrf2. (**C**) Quantitative analysis showing cytosolic Nrf2 and HO-1 normalized to β-actin, and nuclear Nrf2 normalized to Lamin B. All experiments were performed in triplicate (*n* = 3) and data are presented as mean ± SD.

As shown in Fig. 6A and 6B, treatment with KCE significantly upregulated nuclear Nrf2 and the cytosolic antioxidant enzymes HO-1 and NQO1 in a dose-dependent manner in UVB-exposed HaCaT keratinocytes. These results underscore the antioxidant potential of KCE via activation of the Nrf2-mediated defense pathway. Quantitative analysis of HO-1, NQO1, and Nrf2 (Fig. 6C) indicated a dose-dependent recovery of expression following KCE treatment.

## Discussion

Numerous studies have demonstrated the UV protective properties of seaweed extracts, highlighting their potential for usage as treatments to prevent skin damage. Brown seaweed has garnered significant interest in the scientific community recently due to its various bioactive components including polysaccharides, phenolic compounds, pigments, and sterols which contribute to its health benefits and potential uses in cosmetics, pharmaceuticals, and nutraceuticals [20]. *K. crassifolia*, a brown seaweed that is mainly distributed in the coastal areas of Japan [21] possess numerous bioactive properties, such as antioxidant, antitumor, immunomodulatory, and hepatoprotective effects [14, 15, 21, 22]. While previous studies have primarily investigated fucoidan or methanol fractions from this species [14, 15], the present study provides new insights into the photoprotective mechanisms of the ethanol extract of KCE against UVB-induced oxidative stress in human keratinocytes.

The radical scavenging capacity of KCE observed in the DPPH and ABTS^+^ assays reflects its strong antioxidant potential. The percentage inhibition of the ABTS^+^ radical cations determines the decolorization of the ABTS^+^ extent. DPPH is an effective reagent for examining the free radical scavenging properties of KCE and has been widely utilized as a free radical to investigate reducing substances [18, 23]. The ethanolic extract exhibited stronger radical scavenging activity in the DPPH assay, suggesting higher efficacy against lipophilic than hydrophilic radicals. This difference likely reflects the extract’s composition, as phenol and sterol-type compounds show greater reactivity in lipophilic systems. The weaker ABTS activity may result from limited solubility and aqueous-phase stability of active constituents. These findings highlight the polarity-dependent antioxidant potential of the extract and suggest greater relevance in mitigating lipid peroxidation compared to aqueous-phase oxidative processes. Collectively, these results highlight the extract’s direct radical scavenging potential under cell-free conditions.

Beyond this chemical capacity, KCE is expected to exert regulatory effects on intracellular redox homeostasis under oxidative stress. At the cellular level, KCE significantly reduced UVB-induced ROS generation and apoptotic cell death in HaCaT keratinocytes, highlighting its protective capacity under oxidative stress conditions. UVB has been shown to penetrate the stratum corneum, leading to the production of ROS in the underlying layers of viable cells, and causing apoptosis and inflammatory reactions [24, 25]. Interestingly, KCE exhibited a protective effect against oxidative stress via diminishing intracellular ROS production in UVB-exposed HaCaT keratinocytes in a dose-dependent manner.

Furthermore, UVB-induced oxidative stress is a major factor in the complex process of skin apoptosis, which involves a range of molecular mechanisms including DNA damage, apoptotic body formation, and the activation of pro-apoptotic proteins [24]. As reported, sub-G_1_ apoptotic cell populations increased with UVB exposure compared to the control, and dose-dependently reduced with low molecular weight fucoidan from *Sargassum horneri* [25]. KCE appears to act through intracellular signaling regulation, reflecting a distinct composition and mode of action. The suppression of mitochondrial-mediated apoptosis by KCE, evidenced through decreased pro-apoptotic protein expression and increased anti-apoptotic signaling, suggests a regulatory effect on mitochondrial integrity. This is particularly noteworthy given that mitochondrial dysfunction is a central event in UVB-induced skin damage. The presence of lipophilic bioactives in KCE may stabilize mitochondrial membranes and inhibit the release of apoptogenic factors such as cytochrome c, which in turn prevents activation of downstream caspases.

Furthermore, KCE attenuated UVB-induced phosphorylation of MAPK and NF-κB signaling pathways, which are key mediators of oxidative stress and inflammation. Oxidative stress caused by UVB activates the upstream signaling pathways, including MAPK and NF-κB, which trigger inflammation [9]. The phosphorylation of NF-κB and MAPK signaling and NF-κB p65 nuclear translocation were notably downregulated by KCE in UVB-exposed HaCaT keratinocytes in the present study. It suggests that KCE not only limits oxidative damage but also modulates inflammatory signaling cascades. These findings are in line with earlier observations from other brown seaweed extracts [26], yet the simultaneous suppression of both MAPK and NF-κB activation by KCE has not been reported, emphasizing the broad mechanistic potential of KCE.

As indicated in previous studies, antioxidant enzymes play a key role in cells by reducing oxidative stress [16, 24, 27, 28]. The current observations suggested that the cytoprotective effect of KCE on UVB-exposed HaCaT keratinocytes is also mediated via the activation of the Nrf2/HO-1 signaling pathway. Although the present study highlights the pivotal role of the Nrf2/HO-1 pathway in mediating the antioxidant and cytoprotective effects of KCE, it is plausible that additional redox-regulating mechanisms, such as the PI3K/Akt, AMPK, or SIRT signaling pathways, may also contribute to the observed effects. Future investigations focusing on these complementary pathways will be valuable to fully elucidate the molecular network underlying KCE-mediated photoprotection.

Despite these promising findings, several limitations should be acknowledged. First, this study was conducted exclusively using an *in vitro* HaCaT keratinocyte model, which does not fully capture the complexity of human skin, including dermal–epidermal interactions and systemic metabolic processes. Second, since KCE is a crude ethanol extract, the bioavailability and skin penetration of its active compounds remain uncertain, which may affect its efficacy *in vivo*. Third, *in vivo* validation and clinical assessment were not performed, and thus, the translatability of the current results to actual skin physiology requires further investigation. Future studies should focus on the evaluation of skin permeation efficiency, and assessment in animal or human skin models to confirm photoprotective efficacy and safety.

## Conclusion

This study demonstrates, for the first time, that the KCE confers significant protection against UVB-induced oxidative stress and apoptosis in HaCaT keratinocytes. KCE suppressed MAPK and NF-κB signaling while activating the Nrf2/HO-1 antioxidant pathway, indicating a dual regulatory effect on oxidative and inflammatory processes. These findings suggest that ethanol-soluble bioactives play a crucial role in skin photoprotection. Although the study provides compelling mechanistic evidence *in vitro*, further research is required to evaluate skin penetration and validate efficacy through *in vivo* and clinical investigations.

## Supplemental Materials

Supplementary data for this paper are available on-line only at http://jmb.or.kr.



## Figures and Tables

**Table 1:** Yield, total carbohydrate content, crude protein content, and total phenolic compound content of KCE.

Sample	Yield (%)^1^	Carbohydrate (%)^1^	Crude protein (%)^1^	Total phenolic compounds (%)^1^
KCE	25.00 ± 0.13	5.48 ± 0.21	4.77 ± 0.16	2.13 ± 0.01

^1^Average value was present on a dry basis, mean SEM (all experiments were performed in tripli-cate (n = 3)) to determine the repeatability.
